# Knowledge and Attitude of Sciatica Pain and Treatment Methods among Adults in Saudi Arabia

**DOI:** 10.1155/2022/7122643

**Published:** 2022-08-29

**Authors:** Majdi Hashem, Reem Abdulrahman AlMohaini, Norah Ibrahim AlMedemgh, Sara Abdulmajed AlHarbi, Lena Saleh Alsaleem

**Affiliations:** College of Medicine, Al-Imam Mohammad Ibn Saud Islamic University, Riyadh, Saudi Arabia

## Abstract

**Background:**

Sciatica is a relatively common condition, with a lifetime incidence varying from 13% to 40%. The corresponding annual incidence of an episode of sciatica ranges from 1% to 5%. The exact cause of sciatica is unknown to this day; treatment methods and practices differ between individuals based on their cultural background, socioeconomic status, and religious beliefs. This study aimed to assess the knowledge and attitude toward sciatica pain among adults in Saudi Arabia.

**Methods:**

A cross-sectional study was conducted among the adult population in Saudi Arabia. A self-administered questionnaire was distributed among the study population using an online survey. Questions were divided into four groups, each containing multiple questions covering the following aspects: demographic data, past medical history, and the assessment of knowledge and attitudes regarding sciatica.

**Results:**

A total of 3,764 respondents were involved in this study, with an age range of 18–65 years old (females 59.8%). The mean knowledge score was 3.8 (SD 2.1), with the majority having poor knowledge (60.1%). The most common source of sciatica information was an orthopedic or a neurosurgeon, while the most common self-treatment used was painkiller medications (30.8%). The mean attitude score was 35.8 (SD 5.3), with most respondents having a neutral attitude (80.3%). The factors correlated with an increase in knowledge and attitude were having a bachelor's or higher degree and living in an urban area.

**Conclusion:**

While the attitude of the adult population toward sciatica pain seems adequate, their knowledge was shown to be deficient. Furthermore, when comparing diagnosed individuals living in cities with those in rural areas, both knowledge and attitudes were shown to be better in individuals living in cities. Awareness programs by health institutions and healthcare professionals are needed to enhance patients knowledge. Various media can be utilized to enhance patients knowledge including social media platforms.

## 1. Introduction

The sciatic nerve (L4 and 5; S1, 2, and 3) is the largest branch of the sacral plexus and the largest nerve in the body as it consists of two main branches, the tibial, and the common peroneal nerves, bound together with fascia [1, 2]. A common condition affecting this nerve is sciatica, also known as lumbar radicular pain (LRP), a debilitating neurological condition caused by a pathology affecting the sciatic nerve or root that can develop suddenly with physical activity or gradually; according to available data from fundamental science and clinical studies, a combination of inflammation and compression is necessary for the nerve root to be symptomatic [3–8]. Sciatica is a relatively common condition, with a lifetime incidence varying from 13% to 40%. The corresponding annual incidence of an episode of sciatica ranges from 1% to 5% [6, 8, 9]. It is characterized by severe pain that originates in the hip joint, radiates down the leg, and ends at the toe. In most situations, paresthesia, or numbness of the leg when standing or walking, goes hand in hand with leg discomfort and a burning sensation deep in the buttocks. People with this condition will have their pain aggravated by twisting, bending, or coughing [3–5, 10–12]. Several pathological processes contribute to sciatica or lumbar radicular pain (LRP). The commonest underlying cause is the compression of the sciatic nerve at the level of L4/L5 and is generally caused by disc herniation; other causes include disc prolapse, lower back muscle spasms, loss of lumbar lordosis, lifting large weights at incorrect angles, and carrying heavy loads. Wearing one's wallet in one's hip pocket, adopting incorrect sitting and standing positions, and lacking sufficient vitamin D and calcium are all contributing factors for the development of sciatica [3, 4, 10, 12]. Sciatica is a reasonably frequent ailment in pregnancy as it is usually reversible and does not need therapy. This condition occurs because fetal weight compresses the sciatic nerve during sitting or leg muscle spasms. It also causes leg numbness, leading to falls and loss of balance [10]. Although the literature does not address sciatica in Saudi Arabia, we believe it is a common complaint among Saudi Arabians. The literature shows that low back pain, in general, is a leading cause of long-term disability worldwide [13]. The rapid socioeconomic change in Saudi Arabia (SA) has resulted in new public health challenges and extensive health reforms. One of these is low back pain; the prevalence in Saudi Arabia is estimated to range between 63.8 and 89% [14]. A previous qualitative interview study was conducted to assess understanding sciatica illness and treatment beliefs in a lumbar radicular pain population in the United Kingdom (UK). Another study that was conducted in Saudi Arabia explored knowledge around back pain and spinal disorders among Saudi patients in 2016. According to the study, most patients with lower back pain had limited knowledge about their condition [15]. As a result, the primary objective of this study was to analyze the knowledge and attitude toward sciatica pain among Saudi adults. Our secondary objectives were to identify and recognize common misunderstandings about the disease and estimate the prevalence of sciatica among adults in Saudi Arabia.

## 2. Patients and Methods

### 2.1. Study Design and Population

A descriptive cross-sectional study was conducted using an online-administered questionnaire distributed via. online platforms.

The study included all individuals older than 18 years, living in Saudi Arabia, and willing to participate. However, patients who were blind, noncommunicative, intellectually disabled, or severely demented, and were under the age of 18 or lived outside of Saudi Arabia were excluded. The data were collected first by administering the survey to patients who fulfill the inclusion criteria. The study's inclusion criteria were clearly stated at the cover letter of the questionnaire. Participants were asked to involve in the study if they meet the inclusion criteria.

### 2.2. Questionnaire Tool

The instrument included 28 questions and was derived from an intensive literature review. The questionnaire was translated into Arabic and validated by running a pilot test on twenty random participants to recognize any issues related to context and content. The participants received the study questionnaire, which was translated into Arabic (with no specific dialect). To make it more understandable to the general public, we employed a conceptual translation technique rather than a literal (word-for-word translation). To make the questionnaire more understandable to the intended demographic, we used standard the Arabic language (no specific dialect) and translated the items of the questionnaire without using any technical or vernacular phrases. The translation was checked by bilingual specialists in the subject who are fluent in both Arabic and English. The questionnaire included the participant's demographics, educational background, and questions related to sciatica. All participants received written consent before completing the questionnaire. After reading the study's cover letter, participants were asked whether they agreed to give their consent and participate in the study before completing the questionnaire. Participants who gave their consent were instructed to complete the questionnaire; otherwise, their participation was canceled. We had healthcare specialists from several relevant disciplines to examine the face validity of the questionnaire for clarity and comprehensibility and they affirmed that it would be easily comprehended based on their experience.

### 2.3. Ethical Approval

The study was approved by the research ethics committee at Al-Imam Mohammad Ibn Saud Islamic University, Riyadh, Saudi Arabia (HAPO-01-R-011/23-08-2021), and it follows the National Committee of Bio-Ethics guidelines.

### 2.4. Statistical Analysis

The data analyses were carried out using the statistical package for the social sciences, version 26 (SPSS, Armonk, NY: IBM Corp.). The knowledge of sciatica pain and management was obtained by calculating the total score of the 9-item knowledge questionnaires discussed in Table 1, where correct answers were identified and coded as 1, while incorrect answers were coded as 0. The total knowledge has been calculated by adding all 9 items. A score range from 0 to 9 points has been generated. By using 50% and 75% as cutoff points to determine the level of knowledge, participants were classified as having poor knowledge if the score was below 50%, 50% to 75% was considered as moderate knowledge, and above 75% the score was considered good knowledge. For attitude, this has been assessed by 11-item questionnaires (discussed in Table 1) which were the 5-point Likert scale type of categories as the answer options ranging from “strongly disagree” coded as 1 to “strongly agree” coded as 5. Negative questions were coded reversely to avoid bias in the score. The total attitude score has been calculated by adding all 11 items. The score range produced was from 5 to 55 points. By using 50% and 75% as cutoff points to determine the level of attitude, participants were considered negative if the score was below 50%, 50% to 75% was considered neutral attitude, and above 75% was considered a positive attitude level. Descriptive statistics were presented using numbers, percentages, the mean, and standard deviation. The previous diagnosis of sciatica pain was compared with the sociodemographic characteristics of participants by using the Chi-square test. A *P* value <0.05 was set as the significant level. Furthermore, the knowledge and attitude scores were compared with the sociodemographic characteristics of participants using the Mann–Whitney *z*-test and the Kruskal–Wallis test. Statistical collinearity was calculated by using the Shapiro–Wilk test. Both knowledge and attitude scores follow a nonnormal distribution. Thus, nonparametric tests were applied. A Pearson correlation coefficient test was conducted to determine the correlation between knowledge and attitude scores.

## 3. Results

### 3.1. Patient's Characteristics

In total, 3,764 participants were involved in this study.  Table 2 presents the sociodemographic characteristics of participants. The most common age group was 18–25 years old (41.4%). Nearly, 60% of our study sample consisted of females and majority were Saudis (93%). More than half reported that they had bachelor's degrees (55.4%). Around one-third (29.1%) were nonhealthcare employees and 31.9% were university students. Participants living in the western region were 34.6% and 80.1% of the respondents were residing in urban areas. The most common source of information about sciatica pain and management was a physician (49%).

### 3.2. Epidemiology of Sciatica

**Figure 1:**
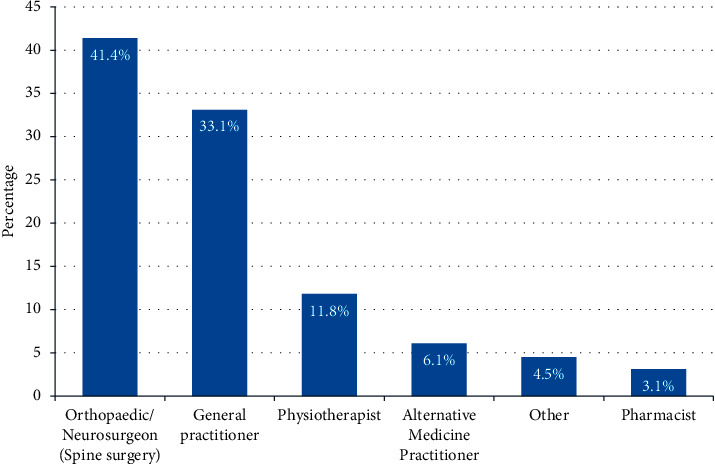
Specialist who provided advice after experiencing sciatica pain.

The overall prevalence of sciatica pain was 13.5%. We found that the prevalence of sciatica pain was more common among those in the age group of 35–49 years (*P* < 0.001), Saudis (*P* < 0.001), those with bachelor's degrees (*P* < 0.001), those who were nonhealthcare providers (*P* < 0.001), and those who indicated a physician is the main the source of information (*P* < 0.001). As indicated in Figure 1, a orthopedic/neurosurgeon was the most commonly reported specialist who provided advice to the respondents after experiencing sciatica pain (41.4%), followed by the general practitioner (33.1%) and physiotherapist (11.8%).

### 3.3. Knowledge and Attitude toward Sciatica Pain

Table 1 shows the assessment of the knowledge and attitude toward sciatica pain. It was revealed that 71.6% believe that sciatica is a pain that radiates from the lower back through the back or sides of the legs. More than half of the study participants (68%) were aware that sciatica symptoms included pain, numbness, tingling sensations extending from the lower back to the toes, and weakness of leg and foot muscles and 65.5% were aware that age, weight, nature of work, and prolonged sitting are risk factors for sciatica. Less than half of the study participants (42%) knew that the most common cause of sciatica was a herniated vertebral disk, which often occurs with age. On the other hand, the knowledge of respondents that physiotherapy and steroid injections are methods to treat sciatica was poor, as only 33% agreed with it. Also, 33% believe that NSAIDs and muscle relaxants are the methods to reduce sciatica. Few of them believe that people with sciatica should avoid movement as it may cause more injury (25.2%), and only 18.8% believe that having sciatica means you will end up with a movement disability. Additionally, only 18.2% believe that sciatica is preventable and may not recur. The overall mean knowledge score was 3.8 (SD 2.1), with poor, moderate, and good knowledge accounting for 60.1%, 37.4%, and 2.5%, respectively. Regarding attitude, the highest agreement to attitude was identified in the following statements: “Regular exercise and proper sitting can significantly contribute to back protection” (mean score: 4.2), followed by the statements,“ Spinal CT/MRI can diagnose sciatica” (mean score: 3.8) and “The severity of pain varies from mild to very severe and it intensifies when sneezing or coughing or after prolonged sitting” (mean score: 3.8), while the least was the statement about “Cupping therapy can reduce or treat sciatica pain” (mean score: 2.8). The overall attitude score was 35.8 (SD 5.3), with negative, neutral, and positive attitudes comprising 4.9%, 80.3%, and 14.7%, respectively.

**Figure 2:**
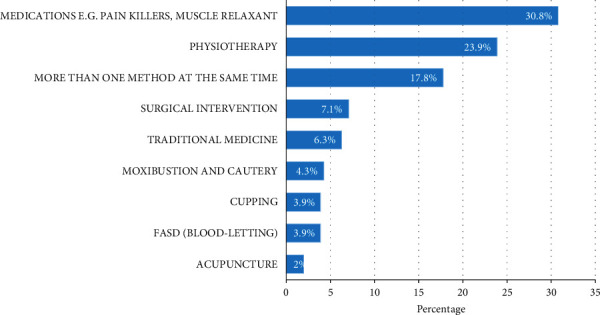
Self-treatment of sciatica.

Figure 2 presents the most commonly reported self-treatment method for sciatica pain. The most common self-treatment method for sciatica pain was pain killers or muscle relaxant medications (30.8%), followed by physiotherapy sessions (23.9%) and a combined method (17.8%).

**Figure 3:**
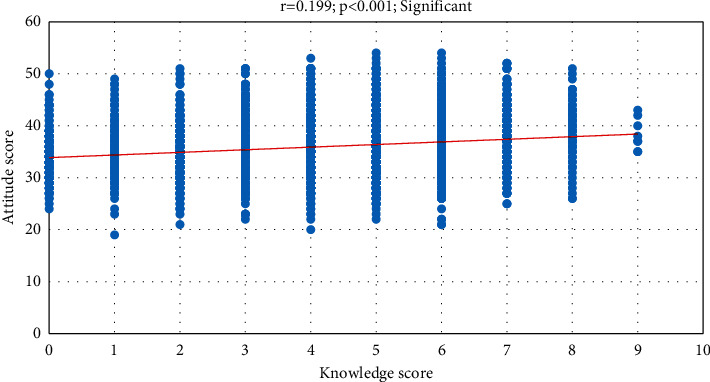
Correlation (Pearson r) between the knowledge score and attitude score.

A significant positive correlation was observed between the knowledge score and the attitude score (*r* = 0.199; *P* < 0.001), Figure 3.

### 3.4. Patient's Characteristics and Their Knowledge and Attitude toward Sciatica Pain

Table 3 presents the mean knowledge and attitude toward sciatica pain. A higher knowledge score was observed among those with bachelor or higher degrees (*Z* = 3.428; *P*=0.001), those living in urban areas (*Z* = 3.85; *P* < 0.001), those who had been diagnosed with sciatica (*Z* = 7.606; *P* < 0.001), and those with a physician as a source of information regarding sciatica pain and management (*H* = 12.809; *P* < 0.05). Furthermore, a higher attitude score was found among respondents with an age group ≤25 years old (*Z* = 7.807; *P* < 0.001), gender female (*Z* = 3.515; *P* < 0.001), Saudi nationality (*Z* = 3.816; *P* < 0.001), bachelor's or higher degrees (*Z* = 2.587; *P*=0.010), university students (*H* = 75.777; *P* < 0.001), those living in the Eastern region (*H* = 41.514; *P* < 0.001), living in the urban area (*Z* = 6.540; *P* < 0.001), those who had not been diagnosed with sciatica pain (*Z* = 3.653; *P* < 0.001), and those who indicated the Internet or books as a source of sciatica pain information (*H* = 161.788; *P* < 0.001).

## 4. Discussion

Till today, the exact cause of sciatica is still idiopathic, and treatment methods and practices vary depending on people's beliefs and practices. Many studies have been conducted to observe people's behaviors while dealing with lower back pain (LBP) and sciatica. Symptoms can be distressing and affect daily life and productivity. Initial treatment aims to manage pain and maintain the function while the compression and/or inflammation subsides [7]. This study aims to determine the knowledge and attitude of the adult population regarding sciatica pain and management. We believe that this is the first study in Saudi Arabia that evaluated the knowledge and attitude regarding sciatica, focusing on the adult population. The outcome of this study could be vital in the literature since it may give insight into how people understand and treat the excruciating pain due to lower back pain (LBP), including sciatica.

In this study, the knowledge regarding sciatica was shown to be poor. Around 60% of the population exhibited poor knowledge levels, while 37.4% were moderate and only 2.5% were considered to have good knowledge (mean score: 3.78); the results go consistently with the paper by Awwad et al. [15]. Based on their study, most of the patients with LBP had limited knowledge regarding their condition.

Another study done by Tavafian et al. [16] found that the knowledge of patients regarding LBP and its risk factors was suboptimal, adding that LBP was not understood by the majority of the patients (74%). However, Lobo et al. [17] revealed better knowledge among adolescents. Accordingly, they found that the knowledge of LBP among adolescents was moderate (45.4%), higher than our results. In our study population, better knowledge is associated with better education, but there was no difference between age and gender. These findings are strikingly similar to the study of Awwad et al. [15], where they documented that the educational level showed a significant relationship with knowledge; however, they found no significant correlation between knowledge and age or gender. In a study published by Lobo et al. [17], they indicated that the presence of sciatica pain did not significantly influence their level of knowledge about the disease. Contrary to this finding, our study revealed that the occurrence of sciatica pain significantly increased the level of the populations' knowledge of the disease. Likewise, the source of sciatica information was another predictor of knowledge where having a physician as a source of information could significantly increase the knowledge level.

A systematic review conducted in Saudi Arabia assessed the prevalence and risk factors for back pain among health workers and found that LBP is highly prevalent among health workers in Saudi Arabia compared with international rates. The most-reported individual risk factors were age, body mass index, and female gender. Occupational factors included bending and twisting backs, lifting objects, and performing hand-to-hand procedures [18]. A previous study done in Portland included 2,945 consecutive patients with ambulatory low back pain of mechanical origin, who were selected to compare the characteristics of patients with chronic low back pain treated by primary care medical (MD) physicians and chiropractic (DC) physicians. It showed that patients treated by MD physicians had lower incomes and were younger; a third party more often funded their care; and their baseline pain and disability were slightly higher. Additionally, patients treated by MD physicians had four times as many visits as those treated by DC physicians. The number of factors determining care sought from primary care medical physicians versus DC physicians may differ depending on the patient's sociodemographics, current pain level, and functional capability. Long-term evaluations indicate that chronic back pain is persistent and difficult to treat by either type of provider [19, 20]. Also, a cross-sectional study about knowledge, attitudes, and beliefs on contributing factors was published in Malawi, which showed that many Malawian patients with chronic pain lack adequate knowledge about chronic pain, and their attitudes and beliefs toward chronic pain are negative. Consequently, LBP management programs in Malawi should include educational programs that instill knowledge about LBP among patients and change their beliefs about their pain. Treatment goals can be achieved more effectively by enhancing the patients' understanding of their pain [19]. Ong and colleagues interviewed thirty-seven people. Their results show that identifying a sciatica cause and being clinically diagnosed allowed people to make sense of it. During the interview, the pain of sciatica was all-encompassing, persistent, and intense. The clinicians' appreciation of this and the provision of clear information about treatment and prognosis were considered crucial. The expectation of pain relief and adverse effects varied among patients, and people carefully balanced their expectations [21]. A study assessed the effectiveness of Hijama for sciatica pain. In total, 67% of patients reported some level of pain relief. Based on their results, Hijama may provide effective an alternative pain relief for most patients by relieving pain and improving their quality of life [10]. Previous systematic reviews assessed prognostic factors predicting the outcome in nonsurgically treated patients. There was no relationship between most of the factors evaluated, including age, body mass index, smoking, and sensory disturbance. As a prognostic factor for subsequent surgery, leg pain intensity at baseline was the only positive correlation with strong evidence [22, 23].

We noted several statements in the specific knowledge assessment where the subjects exhibited poor knowledge. For instance, only 18.2% of the subjects knew that sciatica is not a preventable disease. They also had little knowledge (18.8%) that sciatica may end up with a movement disability. Similarly, only a quarter (25.2%) knew that limitation of movement is necessary to avoid further injury when having sciatica. In comparison, around one-third (33%) of the population were aware that NSAIDs, muscle relaxants, physiotherapy, and steroid injections are the methods to reduce or treat sciatica pain. Hence, more education is needed to address the insufficient knowledge of sciatica.

The most common methods for diagnosing sciatica include taking a medical history and doing a physical examination. The clinical course of acute sciatica is generally regarded to be favorable [3, 24]. In terms of treatment, in most instances, sciatica goes away on its own; according to the previous literature, one-third of individuals with sciatica recover without therapy within two weeks and three-quarters recover within three months. Despite this, conservative treatment or analgesics to relieve pain, nonsteroidal anti-inflammatory drugs (NSAIDs) reduce inflammation, and rehabilitation can help temporarily relieve discomfort; if the pain does not resolve spontaneously, it may require surgical intervention. However, approximately 10–40% develop chronic pain syndrome, also called “failed back” surgery syndrome [3, 5, 25, 26]. Moreover, most trials and research comparing surgical and nonsurgical therapy for sciatica caused by lumbar disk disease recommend surgery because it provides pain relief sooner [5].

Although the study population's knowledge of Sciatica was limited, their attitude toward it was deemed adequate. 80.3% of them demonstrated a neutral attitude, 14.7% were positive, and fewer than 5% had a negative attitude level (mean score: 35.8). It is comparable to the study of Lobo and colleagues [17], where they reported that 61.9% of adolescents had a good attitude. In our further assessment, several factors were shown to be associated with an increased level of attitude, including being a young adult, female, Saudi Arabian, being a professional, being a university student, living in the Eastern region, residing in the urban area, and has not been diagnosed with sciatica, the Internet, or books as sources of Sciatica's information. Conversely, a significant positive correlation was observed between the knowledge score and attitude score, suggesting that the increase in knowledge is correlated with the increase in attitude. The correlation between knowledge, attitude, and beliefs has been consistently reported by Tarimo and Diener [21]. It can be explained as patients' knowledge regarding their disease is related to the beliefs they possess, and their knowledge could persuade these beliefs of their pain experience [17]. Having a source of information is one way to obtain additional knowledge regarding the subject. In this study, the most frequently mentioned source of information regarding sciatica pain and management was the physician (49%), followed by the Internet or books (31.1%). This is almost consistent with the paper by Tarimo and Diener [20]. They documented that the sources of information for patients with LBP were medical officers, physiotherapists, books, the Internet, the media, and schools. Interestingly, painkillers and muscle relaxants were the most commonly sought medications for the self-treatment of pain. In comparison to a study published by Goldsmith et al. [4], patients with sciatica pain preferred physical activities such as swimming, yoga, stretching, and cycling, which they viewed as being helpful to improve spinal health and to cope with symptoms, but not capable of changing the compression, which was also indicated by Boote et al. [27].

## 5. Conclusion

The attitude of the adult population toward sciatica was considered sufficient; however, the knowledge was shown to be insufficient. Better knowledge and attitudes were noted in already diagnosed, educated adults living in cities. Therefore, healthcare providers need to address the gaps in knowledge to help reduce costs in the healthcare system, especially among the unemployed population. By addressing the gap, educational programs and campaigns can be applied to enhance and fill knowledge and management methods of sciatica in the community. Ultimately, educating the patient on the nature of sciatica, its causes, prevention, and how to treat it may help reduce the healthcare system's cost and let patients know more about their disease, when to seek medical advice, and the red flags related to the disease.

## Figures and Tables

**Table 1:** Knowledge and attitude toward sciatica pain ^(*n* = 3.764)^.

Knowledge statement	Correct answer *N* (%)
(1) The most distinctive sign of sciatica is pain that radiates from your lower back into the back or side of your legs	2695 (71.6%)
(2) Pain, numbness, tingling sensation extending from the lower back down to toes, and weakness of leg/foot muscles are symptoms of sciatica	2559 (68.0%)
(3) Age, weight, nature of work, and prolonged sitting are risk factors of sciatica	2464 (65.5%)
(4) The most common cause of sciatica is a herniated vertebral disc, which often occurs with age	1582 (42.0%)
(5) Physiotherapy and steroid injections are methods to reduce/treat sciatica	1347 (35.8%)
(6) NSAIDs and muscle relaxants are methods to reduce/treat sciatica	1243 (33.0%)
(7) People with sciatica should avoid movement as it may cause more injury^†^	950 (25.2%)
(8) Having sciatica may mean you will end up with movement disability^†^	707 (18.8%)
(9) Sciatica is thought to be preventable and it may not recur^†^	684 (18.2%)
Knowledge score (mean ± SD)	3.78 ± 2.11

Level of knowledge	
(i) Poor	2263 (60.1%)
(ii) Moderate	1407 (37.4%)
(iii) Good	94 (02.5%)

Attitude statement	Mean ± SD
(1) Regular exercising and proper sitting can significantly contribute to back protection	4.15 ± 0.95
(2) Spinal CT/MRI can diagnose sciatica	3.79 ± 0.99
(3) The severity of pain varies from mild to very severe and it intensifies when sneezing or coughing or after prolonged sitting	3.75 ± 0.93
(4) Surgical intervention is the last method to relieve sciatica	3.38 ± 1.14
(5) Traditional therapy is more effective than medical intervention in treating sciatica^†^	3.13 ± 1.21
(6) Drinking turmeric and cinnamon mixed with warm milk can reduce/treat sciatica pain^†^	3.14 ± 1.06
(7) Moxibustion and cautery can reduce/treat sciatica pain^†^	3.03 ± 1.17
(8) FASD (blood-letting) is one of the most effective ways in reducing/treating sciatica^†^	2.95 ± 1.04
(9) Mustard oil massage can reduce/treat sciatica pain^†^	2.85 ± 1.04
(10) Acupuncture can reduce/treat sciatica pain^†^	2.83 ± 1.02
(11) Cupping therapy can reduce/treat sciatica pain^†^	2.77 ± 1.06
Attitude score (mean ± SD)	35.8 ± 5.34

Level of attitude	
(i) Negative	185 (04.9%)
(ii) Neutral	3024 (80.3%)
(iii) Positive	555 (14.7%)

†indicates the correct answer.

**Table 2:** Sociodemographic characteristics of participants in accordance to sciatica pain diagnosed by the physician or alternative medicine practitioner.

Study variables	Overall *N* (%) (*n* = 3764)	Diagnosed with sciatica pain		*P* value^§^
		Yes, *N* (%), (*n* = 510)	No *N* (%) (*n* = 3254)	
*Age group*				
(i) 18–25 years	1559 (41.4%)	108 (21.2%)	1451 (44.6%)	<0.001^∗∗^
(ii) 26–34 years	767 (20.4%)	110 (21.6%)	657 (20.2%)	

*Gender*				
(i) Male	1513 (40.2%)	215 (42.2%)	1298 (39.9%)	0.332
(ii) Female	2251 (59.8%)	295 (57.8%)	1956 (60.1%)	

*Nationality*				
(i) Saudi Arabian	3499 (93.0%)	449 (88.0%)	3050 (93.7%)	<0.001^∗∗^
(ii) Non-Saudi Arabian	265 (07.0%)	61 (12.0%)	204 (06.3%)	

*Educational level*				
(i) High school	1079 (28.7%)	138 (27.1%)	941 (28.9%)	<0.001^∗∗^
(ii) Diploma	375 (10.0%)	83 (16.3%)	292 (09.0%)	
(iii) Bachelor's degree	2086 (55.4%)	244 (47.8%)	1842 (56.6%)	
(iv) Higher education	224 (06.0%)	45 (08.8%)	179 (05.5%)	

*Occupation*				
(i) Unemployed	836 (22.2%)	138 (27.1%)	698 (21.5%)	<0.001^∗∗^
(ii) Healthcare provider	313 (08.3%)	59 (11.6%)	254 (07.8%)	
(iii) Nonhealthcare provider	1095 (29.1%)	173 (33.9%)	922 (28.3%)	
(iv) University students	1199 (31.9%)	50 (09.8%)	1149 (35.3%)	
(v) Retired	321 (08.5%)	90 (17.6%)	231 (07.1%)	

*Residence region*				
(i) Central region	852 (22.6%)	137 (26.9%)	715 (22.0%)	0.118
(ii) Eastern region	774 (20.6%)	104 (20.4%)	670 (20.6%)	
(iii) Western region	1302 (34.6%)	157 (30.8%)	1145 (35.2%)	
(iv) Southern region	424 (11.3%)	59 (11.6%)	365 (11.2%)	
(v) Northern region	412 (10.9%)	53 (10.4%)	359 (11.0%)	

*Place of living*				
(i) Urban area	3015 (80.1%)	394 (77.3%)	2621 (80.5%)	0.083
(ii) Rural area	749 (19.9%)	116 (22.7%)	633 (19.5%)	

*Source of information about sciatica pain and management*				
(i) Physician	1843 (49.0%)	300 (58.8%)	1543 (47.4%)	<0.001^∗∗^
(ii) Alternative medicine practitioner	264 (07.0%)	86 (16.9%)	178 (05.5%)	
(iii) Internet or books	1169 (31.1%)	83 (16.3%)	1086 (33.4%)	
(iv) Social media, friends, and relatives	488 (13.0%)	41 (08.0%)	447 (13.7%)	

§*P* value has been calculated using Chi-square test. ^∗∗^ significant at *P* < 0.05 level.

**Table 3:** Differences in knowledge and attitude scores based on participant socio-demographic characteristics ^(*n* = 3764)^.

Factor	Knowledge		Attitude	
	Score (9) Mean ± SD	*Z*/*H*-test; *P* value	Score (55) Mean ± SD	*Z*/*H*-test; *P*value
*Age group* ^*a*^				
(i) ≤25 years	3.79 ± 2.19	*Z* = 1.224; *P*=0.221	36.6 ± 5.69	*Z* = 7.807; *P* < 0.001^*∗∗*^
(ii) >25 years	3.77 ± 2.05		35.2 ± 4.98	

*Gender* ^*a*^				
(i) Male	3.76 ± 2.15	*Z* = 0.210; *P*=0.833	35.4 ± 5.45	*Z* = 3.515; *P* < 0.001^*∗∗*^
(ii) Female	3.79 ± 2.08		36.0 ± 5.25	

*Nationality* ^*a*^				
(i) Saudi Arabian	3.78 ± 2.10	*Z* = 0.276; *P*=0.783	35.9 ± 5.35	*Z* = 3.816; *P* < 0.001^*∗∗*^
(ii) Non-Saudi Arabian	3.75 ± 2.19		34.5 ± 5.04	

*Educational level* ^*a*^				
(i) Diploma or below	3.62 ± 2.11	*Z* = 3.428; *P*=0.001^*∗∗*^	35.5 ± 5.28	*Z* = 2.587; *P* < 0.001^*∗∗*^
(ii) Bachelor or higher	3.88 ± 2.10		35.9 ± 5.37	

*Occupation* ^*b*^				
(i) Unemployed	3.71 ± 1.99	*H* = 4.406; *P*=0.110	35.2 ± 4.96	*H* = 75.777; *P* < 0.001^*∗∗*^
(ii) Employed	3.78 ± 2.13		35.2 ± 5.17	
(iii) University students	3.84 ± 2.19		36.9 ± 5.68	

*Residence region* ^*b*^				
(i) Central region	3.83 ± 2.06	*H* = 6.175; *P*=0.186	36.1 ± 5.58	*H* = 41.514; *P* < 0.001^*∗∗*^
(ii) Eastern region	3.77 ± 2.17		36.5 ± 5.32	
(iii) Western region	3.72 ± 2.20		35.3 ± 4.92	
(iv) Southern region	3.68 ± 2.07		34.9 ± 5.42	
(v) Northern region	4.01 ± 1.82		36.0 ± 5.82	

*Place of living* ^*a*^				
(i) Urban area	3.85 ± 2.13	*Z* = 4.098; *P* < 0.001^*∗∗*^	36.1 ± 5.40	*Z* = 6.540; *P* < 0.001^*∗∗*^
(ii) Rural area	3.51 ± 1.98		34.6 ± 4.89	

*Had been diagnosed with sciatica by a physician or an alternative practitioner* ^*a*^				
(i) Yes	4.45 ± 1.93	*Z* = 7.606; *P* < 0.001^*∗∗*^	35.0 ± 5.52	*Z* = 3.653; *P* < 0.001^*∗∗*^
(ii) No	3.68 ± 2.12		35.9 ± 5.29	

*Source of information about sciatica pain and management* ^*b*^				
(i) Physician	3.86 ± 2.10	*H* = 12.809; *P*=0.005^*∗∗*^	36.0 ± 5.26	*H* = 161.788; *P* < 0.001^*∗∗*^
(ii) Alternative medicine practitioner	3.74 ± 1.91		32.4 ± 4.18	
(iii) Internet or books	3.79 ± 2.15		36.6 ± 5.41	
(iv) Social media, friends, and relatives	3.47 ± 2.12		34.8 ± 5.19	

^a^ *P*-value has been calculated using the Mann–Whitney *Z*-test. ^b^*P* value has been calculated using the Kruskal–Wallis H-test. ^∗∗^significance is determined at *P* < 0.05 level.

## Data Availability

The data used to support the findings of this study are available from the corresponding author upon request.
